# Retraction: Structure and mechanism of the essential two-component signal-transduction system WalKR in *Staphylococcus aureus*

**DOI:** 10.1038/ncomms14331

**Published:** 2017-02-06

**Authors:** Quanjiang Ji, Peter J. Chen, Guangrong Qin, Xin Deng, Ziyang Hao, Zdzislaw Wawrzak, Won-sik Yeo, Jenny Winjing Quang, Hoonsik Cho, Guan-Zheng Luo, Xiaocheng Weng, Qiancheng You, Chi-Hao Luan, Xiaojing Yang, Taeok Bae, Kunqian Yu, Hualiang Jiang, Chuan He

Nature Communications 7: Article number:11000; DOI: 10.1038/ncomms11000 (2016); Published 03 18 2016: Updated 02 06 2017

In this Article, we presented a structural and functional characterization of the WalKR two-component system for signal transduction and a small molecule that can target WalKR, and reported the identification of amino acids that were proposed to be required for its signal transduction activity. However, it was brought to our attention by Ian Monk, Benjamin Howden, Torsten Seemann and Timothy Stinear in the accompanying Correspondence (Monk *et al.*, 2017) that the mutant strains of *Staphylococcus aureus* that we used for these experiments at the University of Chicago carry additional mutations. Although this error does not invalidate the structure solution presented in Fig. 1 (PDB ID: 4YWZ) and the binding of WalKR by the small molecule DHBP described in Fig. 5a, we can no longer unequivocally conclude that Asp119 and Val149 play an important role in the signal recognition domain of WalKR. We sincerely apologise for any adverse consequences that may have arisen from this error. The authors Quanjiang Ji, Peter J. Chen, Guangrong Qin, Xin Deng, Ziyang Hao, Zdzislaw Wawrzak, Won-Sik Yeo, Jenny Winjing Quang, Hoonsik Cho, Guan-Zheng Luo, Xiaocheng Weng, Chi-Hao Luan, Xiaojing Yang, Taeok Bae, Kunqian Yu, Hualiang Jiang and the corresponding author, Chuan He are retracting the paper as the functional roles of Asp119 and Val149 in the signal recognition domain of WalKR are no longer conclusive. The author Qiancheng You could not be reached for comment.

Monk *et al*. Correspondence: Spontaneous secondary mutations confound analysis of the essential two component system WalKR in *Staphylococcus aureus*. *Nat. Commun*. 8:14403 doi: 10.1038/ncomms14403 (2017).

